# Health Care Professionals' Knowledge, Attitude, and Practice towards Adverse Drug Reaction Reporting and Associated Factors at Selected Public Hospitals in Northeast Ethiopia: A Cross-Sectional Study

**DOI:** 10.1155/2019/8690546

**Published:** 2019-11-30

**Authors:** Belete Kassa Alemu, Tessema Tsehay Biru

**Affiliations:** ^1^Department of Pharmacy, Pharmacology and Toxicology Unit, College of Medicine and Health Sciences, Wollo University, Ethiopia; ^2^Department of Pharmacy, Clinical Pharmacy Unit, College of Medicine and Health Sciences, Wollo University, Ethiopia

## Abstract

**Background:**

The role of health care professionals among other stakeholders in early detection, assessment, documentation, and reporting as well as preventing suspected adverse reactions is very crucial to mitigate drug-related problems in health facilities. Previous reports from literatures have indicated that adverse drug reaction reporting is highly linked to the knowledge and attitude of the health care professionals.

**Objective:**

To assess knowledge, attitude, and practice of health care professionals about adverse drug reactions and the associated factors at selected public hospitals in Northeast Ethiopia.

**Methods:**

A hospital-based quantitative cross-sectional study design was employed. A structured self-administered questionnaire was used to collect data on KAP of selected health care providers by the convenience sampling method. Data were entered into Epi info version 3.5.3 and analyzed using SPSS Version 20. Association between dependent and independent variables was found by using bivariate and multivariate logistic regression analysis where *p* < 0.05 was considered to be statistically significant.

**Results:**

Out of 120 questionnaires distributed, 114 respondents filled and returned, giving a 95% response rate. From total, 49 (43%) were nurses, 26 (22.8%) physicians, 17 (14.9%) pharmacy professionals, 12 (10.5%) health officers, and 10 (8.8%) midwives. About 86 (75.44%) study participants had an inadequate knowledge towards ADR reporting, and half of participants failed to report the adverse drug reactions they encountered. But the majority of participants (84, 73.68%) had a favorable attitude towards ADR reporting. Nurses [AOR = 0.069, 95% CI (0.018–0.275)], health officers [AOR = 0.10, 95% CI (0.015–0.647)], and physicians [AOR = 0.14, 95% CI (0.03–0.64)] were found to be less likely to have adequate knowledge on ADR reporting compared to pharmacy professionals.

**Conclusion:**

Even though the majority of health care professionals had a positive attitude, they had inadequate knowledge and poor practice towards ADR reporting.

## 1. Background

Globally, several numbers of drugs are brought into the market everyday; however, the safety of medicines remains to be a major concern for various population groups due to inadequate knowledge. According to the World Health Organization (WHO), an adverse drug reaction (ADR) is any deleterious, inadvertent and unwanted reaction to drugs when used for prevention, diagnosis, or treatment purposes at therapeutic doses or for modification of physiological malfunction which precludes accidental or deliberate overdosage or drug maladministration [1]. ADRs can be predictable and dose related, unpredictable and nondose related, both dose and time related, time related (delayed reactions), withdrawal reactions, and unexpected reactions due to treatment failure [2,3].

Modern medicines have substantially changed the diseases treatment schemes that improve the treatment outcomes in many medical conditions. However, adverse reactions to medicines are a common cause of morbidity, hospital admissions, longer hospital stay, disability, and even mortality [4]. Not only they have a major impact on public health but they also reduce patients' quality of life and impose a significant financial cost in the health care delivery system [5,6].

The history of ADR monitoring has dated back as much as forty years since the milestone of thalidomide disaster that caused phocomelia in thousands of children in many countries [5,7]. Pharmacovigilance is the science and activity related to the detection, assessment, understanding, and prevention of adverse effects or any other possible drug-related problems [3].

Findings from various studies revealed that ADR reporting is linked to the knowledge and attitude of the health care professionals (HCPs). All HCPs in the country need to alert the Ethiopian Food and Drug Administration (EFDA) about suspected adverse drug reactions in order to facilitate immediate and appropriate actions to be taken to prevent or minimize medicine-related injuries for other patients in the future [2,8].

All ADRs ranging from minor to severe reactions should be reported with particular concern to ADRs to new medicines, serious adverse drug reactions, unexpected reactions, and drug interactions which are potentially serious or clinically significant. In addition, uncertainty of the causal relationship between the drug and ADR should not be a reason for not reporting [2,9].

Underreporting of ADRs by HCPs to the concerned body has long been a big challenge in Ethiopia. Search of literature with regard to knowledge, attitude, and practice (KAP) of HCPs towards ADR reporting in the study areas yielded no results; hence, it is found important to conduct this study to investigate the gaps on KAP of HCPs about ADR reporting and identify factors attributable for inadequate knowledge.

## 2. Methods

### 2.1. Study Area, Design, and Period

A hospital-based quantitative cross-sectional study design was used among HCPs (nurses, medical doctors, pharmacy professionals, midwives, and health officers) working at Kemisse General Hospital (KGH) and Ataye Primary Hospital (APH), Northeast Ethiopia. KGH is located in Northeast Ethiopia, Oromia Special Zone, Amhara National Regional State, 331 km away from north of Addis Ababa. KGH is the only general hospital in a zone that serves 1.2 million populations. APH is located in the North Shewa zone, Amhara National Regional State, 300 km away from north of Addis Ababa and 31 km away from south of Kemisse. The study was conducted from May 1 to May 30, 2019.

### 2.2. Study Population

The study population included all physicians, pharmacy professionals, health officers and nurses, midwives who were working in selected hospitals during the study period.

### 2.3. Variables

- Dependent variables
  - Overall knowledge of HCPs about ADR reporting
  - Overall attitude of HCPs towards ADR reporting
- Independent variables
  - Age, sex, profession, level of education, years of experience, and attending training on ADR reporting and type of hospital (primary or general)

### 2.4. Sampling Technique and Sample Size Determination

Convenience sampling technique was used to select all HCPs who have frequent exposure to the ADR.

### 2.5. Data Collection Tool and Data Collection Process

A structured self-administered questionnaire was used. The questionnaire was developed by modifying the tools from other similar studies and the EFDA guidelines [2, 6, 10–14]. Information about the sociodemographic characteristics of respondents and detailed questions used to assess knowledge, attitude, and practices of respondents was included. Prior to commencing the study, the questionnaire with attached written consent form was distributed to HCPs. Participation of the respondents was entirely voluntary.

### 2.6. Data Entry, Management, and Statistical Analysis

Data were entered into Epi info version 3.5.3, cleaned, and then exported to Statistical Package for the Social Sciences (SPSS) version 20 statistical software for analysis. Both descriptive and analytical statistical tests were done. Bivariate and multivariate binary logistic regression analysis was performed to find association between different independent variables and participants' overall knowledge and attitude towards ADR reporting. A *p* value <0.05 was considered as statistically significant association.

In this survey, knowledge of HCPs about ADR reporting was assessed with twelve questions. Each correct response had a score of 1, and each wrong response had a score of 0. Thus, the total score varies from 0 to 12 points (Table 1). The overall level of knowledge was categorized by using the mean score. Participants who score more than or equal to the mean score were classified as having adequate knowledge, and scores below the mean were classified as having an inadequate knowledge. The participants' attitude was evaluated by using thirteen questions rated on a three-point Likert scale such as agree, neutral, and disagree. A response of “agree” was given a score of 3, “neutral” a score of 2, and “disagree” a score of 1, and a score more than or equal to 75% was indicative of a favorable attitude, and a score below 75% would be indicative of unfavorable attitude towards ADR reporting. HCPs' practice was assessed by identifying whether they documented and reported ADRs or not.

### 2.7. Data Quality Control Measures

After the data collection format was prepared, pretest of the tool was done on 10 HCPs working at Dessie Referral Hospital for any necessary amendments to check its suitability for the actual data collection. And, the data were cleared and checked everyday for completeness and consistency before data processing and analysis.

  Adequate knowledge on ADR reporting: those who had a score of more than or equal to a mean score out of 12 questions used to assess knowledge of the respondents
  Favorable attitude towards ADR reporting: those who had a score of more than or equal to 75% response (score ≥ 29.25 on 3-point Likert scale) of the total 13 questions used to assess attitudes of the respondents
  Good practice: those who documented and reported the encountered ADRs
  Inadequate knowledge: those who had less than 6 correct response of the total 12 questions
  Poor practice: those who did not document and report the encountered ADRs
  Unfavorable attitude: those who had less than 75% response (score < 29.25) of the total 13 questions

## 3. Results

### 3.1. Sociodemographic Characteristics

The study was conducted in KGH and APH among 114 HCPs to assess KAP towards ADR reporting. From 120 self-administered questionnaires distributed, 114 were adequately filled and returned within the stipulated time frame giving 95% response rate. Among 114 respondents, 72 (63.2%) and 42 (36.8%) were males and females, respectively. The mean age of respondents was 27.54 (±3.88), and most of them (81, 71.05%) were in the age range of 25–34 years. Most of the respondents (49, 43. 0%) were nurses followed by physicians (26, 22.8%), pharmacy professionals (17, 14.9%), health officers (12, 10.5%), and midwives (10, 8.8%). Most respondents (95 (83.3%)) did not take training on ADR reporting (Table 2).

### 3.2. Knowledge and General Awareness of HCPs on ADR Reporting

Twelve different questions were used to assess knowledge of HCPs on ADR reporting. 100 (87.7%) respondents knew that all drugs available in the market are not safe, and 76 (66.7%) were able to differentiate ADR from overdose toxicities. Only 23 (20.2%) respondents knew the term pharmacovigilance and understood its function. Likewise, 24 (21.1%) and 26 (22.8%) respondents knew the availability of national reporting system and ADR reporting form in Ethiopia, respectively. 35 (30.7%) respondents knew the responsible body that monitor ADRs in Ethiopia. Less than half of respondents (55 (48.2%)) knew that ADR reporting is a professional obligation. 25 (21.9%) respondents replied that the possibility of an ADR should be the first differential diagnosis at all times in patients taking medicines. Moreover, significant proportion of the respondents (93 (81.6%) and 52 (45.6%)) replied that ADRs should be reported only when they are serious and life-threatening and severe and cause disability, respectively (Table 1).

When the general awareness of respondents was assessed, the study has found that more than half of the respondents (64 (56.1%)) were using National Drug Formulary and Standard Treatment Guideline (STG) followed by standard text books (53 (46.5%)) as the main sources of information about ADR. 37 (32.5%) and 32 (28.1%) of the respondents responded that ADRs should be reported to EFDA and Drug and Therapeutic Committee (DTC) of the respective health facility, respectively. Prescribing error, dispensing error, life style of the patient, overdose, and nonadherence are the possible factors that predispose a patient to ADR in 92 (80.7%), 87 (76.5%), 86 (75.4%), 79 (69.3%), and 55 (48.2%) respondents, respectively (Table 3).

### 3.3. Attitude of HCPs towards ADR Reporting

Regarding the attitude of HCPs towards ADR reporting, 100 (87.7%) respondents agreed that ADR reporting should be part of their duty and 87 (76.3%) supported that ADR reporting should be compulsory and 84 (73.7%) agreed that one report of ADR makes a difference. Besides, most respondents 108 (94.7%) and 101 (88.6%) agreed that reporting ADR is important for the public and improves quality of patient care, respectively. 88 (77.2%) health professionals agreed that ADRs should be reported spontaneously at a regular base with 87 (76.3%) emphasizing that there should be certainty for ADRs related to the drug before reporting. Approximately half of respondents (60 (52.6%)) worried about legal problems while ADR reporting (Table 4).

### 3.4. Practice of HCPs regarding Identification, Recording, and Reporting of ADRs

The present study has found that only a small number of respondents (34 (29.82%)) encountered at least one patient with ADR in the past 12 months of their clinical practice, out of which 24 (70.59%) and 17 (50%) respondents recorded and reported ADRs, respectively. From those who have reported ADRs, 8 (47.06%) respondents reported to hospital and pharmacy department and 5 (29.41%) respondents to EFDA. Although about half of the respondents (59 (51.8%)) preferred yellow card for ADR reporting, most of them did not use it regularly due to unavailability of the form. In addition, 44 (38.60%) HCPs have not been usually given proper advice to their patients on possible adverse effects of drugs they prescribed, dispensed, or administered (Table 5).

### 3.5. HCPs' Reasons for Not Reporting ADRs and Suggested Strategies to Improve ADR Reporting

On a 5-point Likert scale evaluation of the reasons for not reporting ADRs, respondents agreed that lack of feedback (67 (58.8%)), reporting forms are not available when needed (53 (46.4%)), not knowing where to report (53 (46.4%)), not knowing how to fill and report the report form (47 (41.2%)), other colleagues are not reporting ADR cases (43 (37.7%)), and uncertain that causal association between the drug and ADR (41 (35.9%)) were the leading discouraging factors contributing to underreporting in hospitals (Table 6).

**Figure 1:**
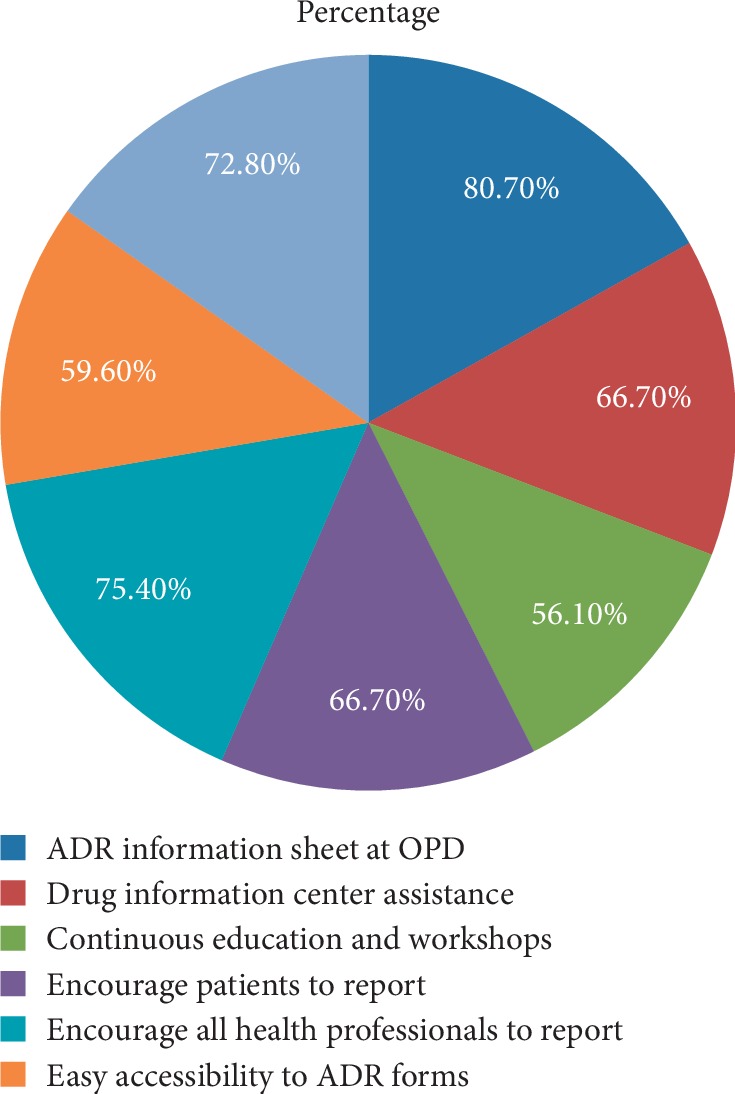
Perceived strategies suggested by HCPs to enhance ADR reporting.

The most common strategic approaches suggested by the respondents to foster ADR reporting were availability of ADR information sheets at OPD (92 (80.7%)), encouraging all health professionals to report (86 (75.4%)), training to report ADR (83 (72.8%)), encouraging patients to report (76 (66.7%)), drug information center assistance (76 (66.7%)), and easy accessibility to ADR forms (68 (59.6%)) (Figure 1).

### 3.6. Factors Associated with HCPs' Knowledge of ADR Reporting

Bivariate logistic regression analysis was run to identify any association between different independent variables and knowledge of HCPs on ADR reporting. Accordingly, attending training on ADR reporting and type of profession were found to have statistically significant association with knowledge. Participants who had not taken training on ADR reporting were 0.722 times (72.2%) less likely to have adequate knowledge compared with participants who had taken training on ADR reporting [COR = 0.278, 95% CI = 0.099–0.779]. Also, nurses, health officers, physicians, and midwives were 94.2% [COR = 0.058, 95% CI = 0.015–0.224], 91.7% [COR = 0.083, 95% CI = 0.013–0.526], 90.1% [COR = 0.099, 95% CI = 0.024–0.414)], and 82.1% [COR = 0.179, 95% CI = 0.032–0.985] times less likely to have adequate knowledge on ADR reporting compared with pharmacy professionals, respectively. Multivariable binary logistic analysis was also run to look into associations controlling any potential confounders. Accordingly, only the type of profession was found to have statistically significant association with knowledge. Nurses, health officers, and physicians were 93.1% [AOR = 0.069, 95% CI = 0.018–0.275], 90% [AOR = 0.10, 95% CI = 0.015–0.647], and 86% [AOR = 0.14, 95% CI = 0.03–0.64] times less likely to have adequate knowledge on ADR reporting compared with pharmacy professionals, respectively. However, none of these variables were found to associate with attitude of HCPs towards ADR reporting (Table 7).

## 4. Discussion

Since ADRs are an important cause of morbidity and mortality and increased health care costs, all HCPs should be alert and keen towards any unexpected or suspected reactions occurring in patients taking medicines, assessing, managing, and reporting the encountered adverse events, which are an integral part of the pharmaceutical care process [2,8,15].

**Figure 2:**
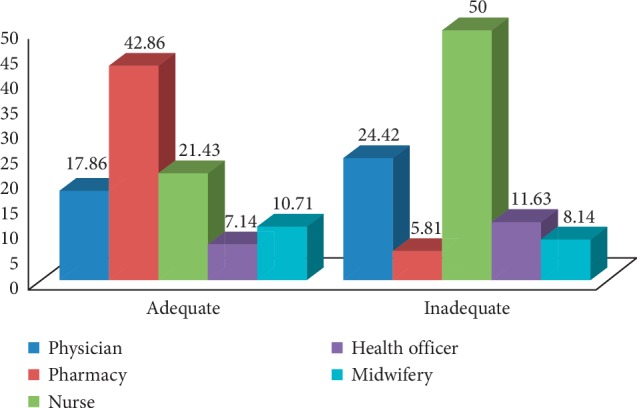
Overall knowledge of HCPs about ADR reporting in selected public hospitals, Northeast Ethiopia, May 2019.

In the present study, the knowledge of HCPs on ADR reporting was low with only 24.56% of the HCPs having adequate knowledge (Figure 2). This result was consistent with similar studies; 21.1% respondents in Tikur Anbessa Specialized Hospital (TASH), Addis Ababa [14], 23.17% in Southwest Ethiopia [16], 33.33% in Northeast Ethiopia [17], and 34.2% in Amhara region, Ethiopia [12], had adequate knowledge about ADR reporting. But this finding was lower than other similar studies; 39.4% of HCPs in Nepal [18], 39.6% in Saudi Arabia [19], 48.2% in Nekemte town, Ethiopia [6], 53% in Gondar town, Ethiopia [10], and 77% in Philippines [20], had adequate knowledge.

In the current study, 66.67% respondents said ADR is different from overdose toxicities or side effects. This figure is higher than a study conducted in Gondar town (64.7%) [10] and Addis Ababa (53. 5%) [14]. However, this result is lower than a result reported by pharmacy professionals in Addis Ababa (85.2%) [21]. But the WHO recommended that the term side effect refers to minor effects related to the pharmacological properties of the drug [7, 22]. In addition, 20.18% of the respondents knew the term pharmacovigilance and its activities which are comparable with a study done in the Jimma zone (19.5%) [16]. Moreover, the present study indicated that a small number of respondents knew the availability of national reporting system (22.81%) and ADR reporting form (21.05%), which is lower than 40.4% in a study done in Lagos, Nigeria [23,24], 49.02% in Gondar [10], and 63.4% at TASH, Addis Ababa [14].

Besides, 58.77% of respondents were aware that all HCPs are responsible and an obligation to report ADRs to the concerned body in contrast to 81.1% in a study conducted in the Amhara region [12]. Moreover, 21.93% of the HCPs said that the possibility of ADR is the first differential diagnosis at all times in patients taking medicines opposed to a report by EFDA, the regulatory agency of Ethiopia [2]. One possible explanation for insufficient knowledge of respondents in this study was that only 19 (16.67%) respondents had taken training on ADRs and their reporting. Hence, creating awareness, providing training, and upgrade knowledge of HCPs about how ADRs are the priority causes of morbidity and mortality and the essence of ADR reporting to the public is essential.

Significant proportion of the respondents (81.58%) replied that ADRs should be reported only when they are serious and life-threatening, while a few respondents (21.05%) were aware that mild to moderate unexpected, certain, and suspected reactions need to be reported. In contrast, a study done in one of the tertiary centers in Northern Nigeria showed that a majority (>70%) of the respondents were aware that suspected, serious, and certain reactions should be reported [25] and 48.6% in a study conducted in Amhara [12]. ADRs are the most ignored but yet constitute the major drug therapy problems and the most important ones to cause major impact on public health, reducing patients' quality of life and a considerable financial burden, and hence, their reporting deemed to be very essential.

**Figure 3:**
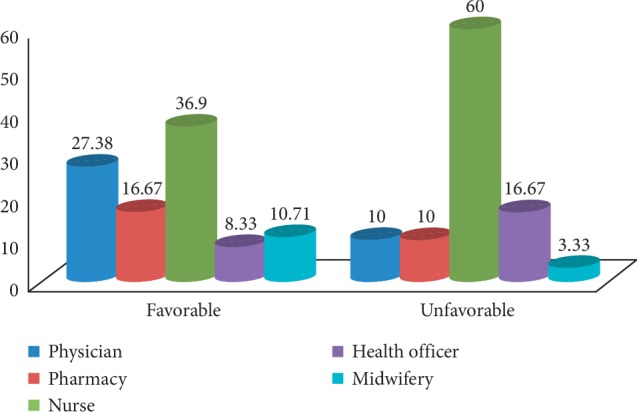
Overall attitude of HCPs about ADR reporting in selected public hospitals, Northeast Ethiopia, May 2019.

Despite the inadequate knowledge of the HCPs, the majority 73.68% had favorable attitude towards ADR reporting (Figure 3), which is higher than reports from other studies; 26.9% in Malaysia [26], 42.1% in Nekemte town, West Ethiopia [6], 52.1% in TASH, Addis Ababa [14], and 66.3% in Nepal [18]. But the present finding is lower than other reports; 82.2% in south India [27], 86% in Gondar town, North Ethiopia [10], 86% in Boru Meda hospital [17], and 90% in other parts of India [28] had positive attitude about ADR reporting. This study showed that 87.7% of respondents agreed that ADR reporting should be part of their duty, which is in line with the findings from similar studies in Addis Ababa, 84% [14], Gondar, 84.3% [10], but higher than a study in Jimma, 57.31% [16]. In addition, 76.3% respondents agreed that affirmation of ADRs related to the drug is necessary before reporting in contrast to 83.3% respondents in a study conducted at Gondar [10]. This perceived obligation to establish causal relation and feeling of legal problems might be one reason for the low reporting rates. Considerable number of respondents (73.7%) agreed that ADR reporting is time-consuming activity and about half (52.6%) of HCPs believed that ADR reporting creates additional workload. This perception of the respondents may substantially affect their motivation of ADR reporting in their clinical practice.

**Figure 4:**
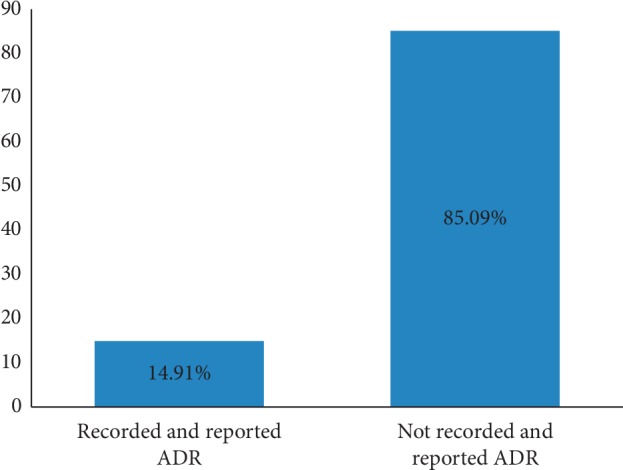
Overall practice of HCPs on ADR reporting in selected public hospitals, Northeast Ethiopia, May 2019. Out of 114 HCPs, only 17 (14.91%) participants had documented and reported encountered ADRs. However, 97 (85.09%) participants did not encounter, document, and/or report ADRs.

Looking at the practice of HCPs about ADR reporting, the present study revealed that 29.82% (Figure 4) in contrast with 38% in TASH, Addis Ababa [14], 55.9% in Gondar [10], 65% in Turkish [29], and 81% in Northern Nigeria [25] encountered patients with ADR in their clinical practice in the past 12 months. Among those HCPs who encountered ADRs, 50% claimed that they have reported the ADR despite only 29.41% reported to appropriate body EFDA. Underreporting of ADRs by HCPs remains to be a challenge in Ethiopia. Not only the health professionals working in hospitals but also the medicine regulatory agencies have to work jointly to monitor drug safety issues and maintain a balance between medicines' benefits and risks.

The major reasons for not reporting of ADRs were lack of feedback; reporting forms are not available when needed, not knowing where to report, not knowing how to fill and report ADR, uncertain of the causal association between the drug and ADR, and insufficient clinical knowledge, among others. Regular sensitization programs such as continued education and workshops and trainings, timely availability of ADR forms and ADR information sheets, feedback, and communication with the concerned bodies (e.g., EFDA) and encouraging all health professionals and patients to report suspected ADRs were some of the strategies forwarded by the respondents to foster ADR reporting (Figure 1). These training programs and continuous feedback clear all misconceptions associated with the ADR reporting as the previous reports have shown significant improvement in knowledge, attitude, and practice of health care workers about ADR after intervention [30–32].

Regarding the factors that affect knowledge of ADR reporting, nurses, health officers, and physicians were 93.1% [AOR = 0.069, 95% Cl (0.018–0.275)], 90% [AOR = 0.10, 95% Cl (0.015–0.647)], and 86% [AOR = 0.14, 95% Cl (0.03–0.64)] times less likely to have adequate knowledge compared with pharmacy professionals, respectively. Similarly, in one study, nurses (*p*=0.001) and health officers (*p*=0.019) had an inadequate knowledge compared to pharmacy professionals [10]. In contrary, a similar study done in the Philippines showed that 86% nurses and 72% of physicians had a good knowledge about ADR reporting and 61% of pharmacists had an adequate knowledge [20].

Conduction of this study was not without limitation. One of the limitations was that we did not include all HCPs working in a hospital, and only those having frequent exposure to drugs and patients were selected. The sample size was small. However, the inclusion of different HCPs does make it valuable. As the study used self-administered questionnaire, recall and personal bias may have affected the data obtained. Above all, it is a simple assessment of KAP in specific hospitals that does not assess multisectors and sort out problems in the area of pharmacovigilance, but without denying that the findings from this study are inputs for further researchers.

From total 114 HCPs, 28 (24.56%) had adequate knowledge, among which pharmacy personnel accounted the majority 42.86%. On the contrary, 86 (75.44%) had inadequate knowledge, of which half of them were nurses followed by physicians (24.42%).

From total 114 HCPs, 84 (73.68%) had favorable attitude about ADR reporting, in which more than half of them were nurses (60%) followed by health officers (16.67%). On the contrary, 30 (26.32%) had unfavorable attitude.

## 5. Conclusion

The present study identified that HCPs working in KGH and APH had inadequate knowledge on ADR reporting and poor documentation and ADR reporting practice which contributed to underreporting in hospitals to EFDA despite the majority HCPs had favorable attitude towards ADR reporting. Hence, taking into account HCPs' suggestions and the study findings, the present study strongly underscore that creating awareness and improving the knowledge of all HCPs through regular sensitization programs, trainings, and timely feedback is a very crucial strategy to enhance spontaneous ADRs reporting in health facilities to the concerned body, which ultimately impacts the provision of quality patient care.

## Figures and Tables

**Table 1:** Health care providers' knowledge of ADR in selected public hospitals, Northeast Ethiopia, May 2019.

Questions	Category	Frequency	Percentage
Know that all drugs in the market are not safe	Yes	100	87.72
	No	14	12.28

Know ADR is different from overdose toxicities/side effects	Yes	76	66.67
	No	38	33.33

Know the term pharmacovigilance	Yes	23	20.18
	No	91	79.82

Write the definition of pharmacovigilance^*∗*^	Yes	10	8.77
	No	104	91.23

Know national ADR reporting system	Yes	24	21.05
	No	90	78.95

Know availability of ADR reporting forms	Yes	26	22.81
	No	88	77.19

Know how to report	Yes	23	20.18
	No	91	79.82

Know the responsible body that monitors ADR in Ethiopia	Yes	35	30.70
	No	79	69.30

Know ADR reporting is a professional obligation	Yes	55	48.25
	No	59	51.75

The possibility of an ADR should be the first differential diagnosis at all times	Yes	25	21.93
	No	89	78.07

Who is the responsible professional to report ADR in hospitals? (yes answers are only indicated)	Medical doctors	84	73.68
	Health officers	77	67.54
	Midwives	68	59.65
	Nurses	75	65.79
	Pharmacy personnel	98	85.96
	All	67	58.77

When should ADRs be reported? (yes answers are only indicated)	Serious and life-threatening	93	81.58
	Severe and cause disability	52	45.61
	Mild and cause less inconvenience	24	21.05

What kinds of ADRs need to be reported? (yes answers are only indicated)	Suspected reactions	50	43.86
	Certain reactions	55	48.25
	Serious reaction, e.g., SJS	70	61.40
	Slight reaction, e.g., nausea	11	9.65
	Reaction to all drugs	31	27.19
	Reaction to new drugs	42	36.84
	Known reactions	20	17.54
	Unexpected reactions	49	42.98
	Drug interactions	33	28.95
	Teratogenic phenomenon	48	42.11

^*∗*^For this item, the correct response was considered when the study participant wrote not only the precise definition but also the general concept of pharmacovigilance. Do not know and unrelated responses were considered as incorrect.

**Table 2:** Sociodemographic characteristics of HCPs in selected public hospitals, Northeast Ethiopia, May 2019.

Variables	Category	Frequency (*n* = 114)	Percentage
Age	<25	25	21.93
	25–34	81	71.05
	≥35	8	7.02

Sex	Male	72	63.16
	Female	42	36.84

Profession	Physician	26	22.81
	Pharmacy personnel	17	14.91
	Nurse	49	42.98
	Health officer	12	10.53
	Midwifery	10	8.77

Level of education	Diploma	20	17.54
	BSC degree	88	77.19
	MSc/MPH	6	5.26

Years of clinical experience	<3	56	49.12
	≥3	59	51.75

Trained on ADR reporting	Yes	19	16.67
	No	95	83.33

**Table 3:** General awareness of HCPs about ADR reporting in selected public hospitals, Northeast Ethiopia, May 2019.

Questions	Frequency	Percentage
*To whom do you think that ADRs should be reported?*		
Manufacturers	17	14.91
MOH	32	28.07
EPA	22	19.30
EFDA^*∗*^	37	32.46
DTC of the respective health facility	19	16.67
Pharmacy department	23	20.18
*Who is primarily responsible to remind and follow-up patients about side effects of drugs they are given?*		
Physicians	74	64.91
Pharmacists	94	82.46
Nurses	80	70.18
Midwifery	66	57.89
Health officers	72	63.16
*What is your source of information about ADR?*		
National drug formulary and STG	64	56.14
Standard text books	53	46.49
Drug sales man	14	12.28
Notes from the training	19	16.67
Search engines (Internet)	23	20.18
Journal articles	14	12.28
Package inserts	24	21.05
Advertisement brochures/leaflets	20	17.54
Direct call to a pharmaceutical company	11	9.65
Pharmaceutical company representative	16	14.04
*What possible factor(s) predispose a patient to ADR?*		
Dispensing error	87	76.32
Prescription error	92	80.70
Overdose	86	75.44
Life style of the patient	79	69.30
Nonadherence	55	48.25

^*∗*^Correct knowledge. MOH: Ministry of Health; EFDA: Food, Medicine, Health Care Administrative and Control Authority; STG: Standard Treatment Guideline; EPA: Ethiopian Pharmaceutical Association; DTC: Drug Therapeutic Committee.

**Table 4:** Attitudes towards ADR reporting among HCPs in selected public hospitals, Northeast Ethiopia, May 2019 (*N* = 114).

Questions	Agree	Disagree	Neutral
Do you feel that ADR reporting can benefit the public health?	108 (94.74)	6 (5.26)	0
Do you feel that ADR reporting improves quality of patient care?	101 (88.60)	10 (8.77)	3 (2.63)
Do you feel that one report can make a difference?	84 (73.68)	18 (15.79)	12 (10.53)
Do you feel that ADR reporting is part of duty of HCPs?	100 (87.72)	12 (10.53)	2 (1.75)
Do you feel that reporting ADR should be compulsory?	87 (76.32)	17 (14.91)	10 (8.77)
Do you feel that only ADR that cause persistent disability should be reported?	48 (42.11)	64 (56.14)	2 (1.75)
Do you feel that ADR reporting is time-consuming activity with no outcome?	27 (23.68)	84 (73.68)	3 (2.63)
Do you feel that ADR reporting creates additional workload?	48 (42.11)	60 (52.63)	6 (5.26)
Do you feel that proper training should be provided to the HCPs for ADR reporting?	99 (86.84)	14 (12.28)	1 (0.87)
Do you feel that confidentiality should be maintained while ADR reporting?	83 (72.81)	24 (21.05)	7 (6.14)
Do you worry about legal problems while you think of ADR reporting	60 (52.63)	39 (34.21)	15 (13.16)
Do you feel that ADRs should be reported spontaneously at a regular base?	88 (77.20)	16 (14.04)	10 (8.77)
Be sure that ADRs are related to the drug before reporting	87 (76.32)	18 (15.79)	9 (7.89)

**Table 5:** ADR reporting practices of HCPs in selected public hospitals, Northeast Ethiopia, May 2019.

Questions	Category	Frequency	Percentage
Have you encountered patients with ADR in the last 12 months?	Yes	34	29.82
	No	80	70.18

How many patients with ADR have you encountered?	One	11	9.65
	Two	8	7.02
	Three	3	2.63
	Four	6	5.26
	Above four	6	5.26

Have you recorded the ADR you encountered on patients clinical record?	Yes	24	70.59
	No	10	29.41

Have you reported ADRs?	Yes	17	50
	No	17	50

If reported, to where did you report that reaction?	Hospital	8	47.06
	MOH	2	11.76
	EFDA	5	29.41
	Pharmaceutical company	1	5.88
	Pharmacy department	8	47.06

Did you have ADR reporting forms?	Yes	29	25.44
	No	85	74.56

How often did you advice patients on possible adverse effects of drugs?	Always	48	42.11
	Usually	22	19.30
	Sometimes	38	33.33
	Never	6	5.26

**Table 6:** Perceived reasons for not reporting ADRs among HCPs in selected public hospitals, Northeast Ethiopia, May 2019 (*N* = 114).

Reasons	Frequency (%)				
	1^a^	2^b^	3^c^	4^d^	5^e^
Concern that the report may be wrong	46 (40.35)	41(35.96)	11 (9.65)	16 (14.04)	0
Not knowing how to fill and report ADR	22 (19.30)	35 (30.70)	12 (10.53)	37 (32.46)	8 (7.02)
Uncertain of causal association between drug and ADR	21 (18.42)	40 (35.09)	12 (10.53)	38 (33.33)	3 (2.63)
Lack of time to fill report form	28 (24.56)	50 (43.86)	11 (9.65)	19 (16.67)	6 (5.26)
Reporting does not influence the t/t scheme	30 (26.32)	46 (40.35)	10 (8.77)	23 (20.18)	5 (4.39)
Forgetfulness	24 (21.05)	32 (28.07)	19 (16.67)	36 (31.58)	3 (2.63)
Lack of feedback	11 (9.65)	22 (19.30)	14 (12.28)	57 (50)	10 (8.77)
Fear of legal liability by reporting ADR	21 (18.42)	38 (33.33)	17 (14.91)	37 (32.46)	1 (0.88)
Concern that a report will generate an extra work	29 (25.44)	42 (36.84)	14 (12.28)	23 (20.18)	6 (5.26)
Belief that only safe drugs are marketed	39 (34.21)	41 (35.96)	11 (9.65)	20 (17.54)	3 (2.63)
Thinking that one report does not make any difference	34 (29.82)	41 (35.96)	11 (9.65)	24 (21.05)	4 (3.51)
Thinking that you may have caused a patient harm	28 (24.56)	51 (44.74)	8 (7.02)	23 (20.18)	4 (3.51)
My report is not needed/necessary	38 (33.33)	52 (45.61)	11 (9.65)	11 (9.65)	2 (1.75)
Insufficient clinical knowledge	22 (19.30)	35 (30.70)	17 (14.91)	38 (33.33)	2 (1.75)
Reporting forms are not available when needed	15 (13.16)	30 (26.32)	16 (14.04)	40 (35.09)	13 (11.40)
Thinking that ADR reporting is not a duty	37 (32.46)	47 (41.23)	4 (3.51)	22 (19.30)	4 (3.51)
Not knowing where to report	18 (15.79)	30 (26.32)	13 (11.40)	42 (36.84)	11 (9.65)
Other colleagues are not reporting ADR cases	19 (16.67)	30 (26.32)	22 (19.30)	39 (34.21)	4 (3.51)

1^a^ = strongly disagree; 2^b^ = disagree; 3^c^ = neutral; 4^d^ = agree; 5^e^ = strongly agree.

**Table 7:** Multivariate analysis of factors associated with knowledge of HCPs in selected public hospitals, Northeast Ethiopia, May 2019.

Variables	Knowledge		COR with 95% CI	AOR with 95% CI
	Adequate (*n* = 28)	Inadequate (*n* = 86)		
*Age (years)*				
<25	8	17	1	1
25–34	20	61	0.697 [0.261–1.857]	
≥35	0	8	0.000	0.000

*Sex*				
Male	20	52	1.635 [0.647–4.130]	
Female	8	34	1	1

*Profession*				
Physician	5	21	0.099 [0.024–0.414]^*∗*^	0.140 [0.030–0.641]^*∗*^
Nurse	6	43	0.058 [0.015–0.224]^*∗∗*^	0.069 [0.018–0.275]^*∗∗*^
Health officer	2	10	0.083 [0.013–0.526]^*∗*^	0.100 [0.015–0.647]^*∗*^
Midwifery	3	7	0.179 [0.032–0.985]^*∗*^	0.230 [0.040–1.336]
Pharmacy	12	5	1	1

*Level of education*				
Diploma	7	13	1.077 [0.156–7.420]	
BSc	19	69	0.551 [0.094–3.329]	
MSc/MPH	2	4	1	1

*Experience*				
<3 years	16	39	1.607 [0.680–3.799]	
≥3 years	12	47	1	

*Training on ADR reporting*				
Not trained	19	76	0.278 [0.099–0.779]	0.451 [0.124–1.637]
Trained	9	10	1	1

*Hospital*				
Primary	16	41	1.463 [0.619–3.458]	
General	12	45	1	

^*∗*^ *p* < 0.05 and ^*∗∗*^*p* < 0.001 (statistically significant).

## Data Availability

All data generated or analyzed during this study are included within the manuscript and supplementary file (STROBE checklist for observational studies). Moreover, the datasets used and/or analyzed (SPSS data) are available from the corresponding author on reasonable request.

## Abbreviations

ADR: Adverse drug reaction
APH: Ataye Primary Hospital
DIC: Drug Information Center
DTC: Drug and Therapeutic Committee
EFDA: Ethiopian Food and Drug Administration
HCPs: Health care professionals
KAP: Knowledge, Attitude, and Practice
KGH: Kemisse General Hospital
MOH: Ministry of Health
SPSS: Statistical Package for Social Sciences
STG: Standard Treatment Guideline
TASH: Tikur Anbessa Specialized Hospital
WHO: World Health Organization.
